# The Framingham Risk Score Is Associated with Chronic Graft Failure in Renal Transplant Recipients

**DOI:** 10.3390/jcm10153287

**Published:** 2021-07-26

**Authors:** Josephine L. C. Anderson, Margot L. Poot, Hannah L. M. Steffen, Daan Kremer, Stephan J. L. Bakker, Uwe J. F. Tietge

**Affiliations:** 1 Department of Internal Medicine, University Medical Center Groningen, University of Groningen, 9713 GZ Groningen, The Netherlands; j.l.anderson@umcg.nl (J.L.C.A.); d.kremer@umcg.nl (D.K.); s.j.l.bakker@umcg.nl (S.J.L.B.); 2 Department of Pediatrics, University Medical Center Groningen, University of Groningen, 9713 GZ Groningen, The Netherlands; margotlouisepoot@gmail.com (M.L.P.); h.l.m.steffen@gmail.com (H.L.M.S.); 3 Division of Clinical Chemistry, Department of Laboratory Medicine, Karolinska Institutet, SE-171 64 Stockholm, Sweden; 4 Clinical Chemistry, Karolinska University Laboratory, Karolinska University Hospital, SE-141 86 Stockholm, Sweden

**Keywords:** Framingham risk score, transplantation, dyslipidaemia, chronic graft failure, kidney

## Abstract

Predicting chronic graft failure in renal transplant recipients (RTR) is an unmet clinical need. Chronic graft failure is often accompanied by transplant vasculopathy, the formation of de novo atherosclerosis in the transplanted kidney. Therefore, we determined whether the 10-year Framingham risk score (FRS), an established atherosclerotic cardiovascular disease prediction module, is associated with chronic graft failure in RTR. In this prospective longitudinal study, 600 well-characterised RTR were followed for 10 years. The association with death-censored chronic graft failure (*n* = 81, 13.5%) was computed. An extended Cox model showed that each one percent increase of the FRS significantly increased the risk of chronic graft failure by 4% (HR: 1.04, *p* < 0.001). This association remained significant after adjustment for potential confounders, including eGFR (HR: 1.03, *p* = 0.014). Adding the FRS to eGFR resulted in a higher AUC in a receiver operating curve (AUC = 0.79, *p* < 0.001) than eGFR alone (AUC = 0.75, *p* < 0.001), and an improvement in the model likelihood ratio statistic (67.60 to 88.39, *p* < 0.001). These results suggest that a combination of the FRS and eGFR improves risk prediction. The easy to determine and widely available FRS has clinical potential to predict chronic graft failure in RTR.

## 1. Introduction

The number of patients with end-stage renal disease (ESRD) has been growing unwaveringly over the past decades [1], resulting in a rising demand for donor kidneys. Renal transplant recipients (RTR) already outnumber dialysis patients in many countries [2]. Even after transplantation RTR still face major clinical problems, namely graft failure and a 4–6-fold increased incidence of cardiovascular disease (CVD) [3], the leading cause of death with a functioning graft [4]. Substantial clinical progress has been made over the past decades regarding acute rejection, but clinical advances concerning chronic graft failure stagnated, eventually leading to return to haemodialysis or re-transplantation in substantial numbers of patients [5]. An important contributing factor to chronic graft failure is transplant vasculopathy (TV). Histopathologically, TV represents the build-up of lesions within the graft that closely resemble classic *de novo* atherosclerotic plaque formation [6,7]. Therefore, atherosclerosis negatively impacts RTR in two distinct ways, as classic atherosclerosis underlying CVD events and as de-novo atherosclerosis in the form of TV. 

Due to the post-transplantation increase in cardiovascular risk, there is rather extensive literature concerning CVD prediction in RTR using traditional risk stratification [8,9,10]. In the general population, the Framingham Risk Score (FRS), that considers gender, age, smoking, systolic blood pressure, total cholesterol, and HDL-C, is commonly used [11]. Unfortunately, the CVD risk stratification in renal transplant recipients has been disappointing, seemingly due to the complex aetiology of CVD in this specific patient population [12,13]. This could be because the years that RTR are on haemodialysis prior to transplantation are damaging to the cardiovascular system in specific ways, rendering risk predictors derived from a general population sample, that was essentially CVD free at time of inclusion, inadequate to predict CVD events and mortality in this specific population. 

However, with respect to chronic graft failure, no consensus has been reached regarding prediction models, although there is a clear clinical need for such an approach given the poor 10-year graft survival. A successfully validated risk prediction module would also allow to tailor targeted intervention approaches, e.g., with pharmacotherapy, to prolong graft survival. Although the overall cardiovascular status of RTR is mostly poor, the allograft often has a normal vascular make-up at the time of transplantation. As chronic rejection is frequently accompanied by de novo atherogenesis, we hypothesise that the FRS as a commonly applied cardiovascular risk score could also serve to stratify RTR at risk of developing chronic graft failure.

## 2. Materials and Methods

### 2.1. Study Population

All RTR with a normally functioning renal graft for at least one year who visited the outpatient clinic in the University Medical Centre Groningen (UMCG) between August 2001 and July 2003 were invited to join the study. Exclusion criteria were overt congestive heart failure, endocrine abnormalities except diabetes mellitus, and cancer other than cured skin cancer. Of the 847 eligible patients, 606 opted to participate in the study (72%). The baseline characteristics showed no difference between participants and the original cohort, making it an accurate representation of the whole. Out of the 606 patients, five had to be excluded due to insufficient data to calculate the Framingham risk score, and one patient was lost to follow-up. Patients were followed for a median period of 10 years. The study was approved by the local Medical Ethics Committee (METc2001/039), and is in accordance with the Declaration of Helsinki. All included subjects gave written informed consent. The TxL-IRI Biobank and Cohort Study is registered at ClinicalTrials.gov with identifier NCT03272854.

### 2.2. Measurements and Definitions

Diabetes was defined as a fasting plasma glucose ≥7.0 mmol/L or the use of antidiabetic medication, in accordance with ADA guidelines [14].

After an 8–12 h fasting period, blood was drawn and routine laboratory measurements were conducted, as previously described [15]. Glucose was measured using the glucose-oxidase method (YSI 2300 Stat Plus; Yellow Springs, OH). Total cholesterol (TC) was determined using the cholesterol oxidase-phenol aminophenazone method. HDL cholesterol was measured with the cholesterol oxidase-phenol aminophenazone method on a Technikon RA-1000 (Bayer Diagnostics, Mijdrecht, The Netherlands). Plasma hs-CRP was determined using in-house enzyme-linked immunosorbent assays (ELISAs). Creatinine concentrations were quantified in both urine and plasma using a modified version of the Jaffé method (MEGA AU 510; Merck Diagnostica). Creatinine clearance was computed from 24-h urinary creatinine excretion and plasma estimated glomerular filtration rate (eGFR, calculated using the CKD-EPI formula). Body Mass Index (BMI) and waist circumference were measured, as previously described [15]. We assessed class I and class II human leukocyte antigen (HLA) antibodies by ELISA (LATM20x5, One Lambda, Canoga Park, CA, USA). Samples were classified as positive, borderline, and negative, according to the instructions of the manufacturer.

### 2.3. Endpoints and Outcome Measures

The main predictor of this study was the 10-year Framingham cardiovascular risk score, which was calculated using the 10-year cardiovascular risk algorithm of the Framingham Heart Study [11,16]. The primary endpoint was death-censored graft failure, defined as a return to dialysis therapy or re-transplantation. Time to graft failure was measured from the date of inclusion until occurrence of graft failure or censoring due to death, i.e., deceased participants are not regarded as allograft failure.

### 2.4. Statistical Analysis

Renal transplant recipients were divided into three pre-defined groups based on the FRS, namely “low risk” (<10%), “intermediate risk” (10–20%) and “high risk” (≥20%), as established in earlier studies [17,18]. Differences in baseline characteristics were tested between these groups. Categorical values are given as absolute numbers (percentages), and differences are tested with a chi-squared test. Normally distributed continuous variables are given as mean ± standard deviation (SD) and differences are tested by one-way analysis of variance (ANOVA). Skewed continuous variables are presented as median (25th to 75th percentile) and differences between groups are tested by the Kruskal-Wallis test.

A multivariable Cox regression analysis was performed to calculate the hazard ratio (HR), with 95% confidence intervals (CI), for chronic graft failure. However, the Schoenfeld residuals test was significant, indicating a violation of the proportional hazard assumption (*p* < 0.05). In order to account for the varying effect of the FRS over time, an extended Cox model was used. A time varying covariate (TVC) was computed, therefore rendering the proportional hazards assumption obsolete [19]. The TVC of the FRS was included as an independent variable in all further analyses. Cumulative hazards were used to graphically display the results of the survival analysis. To assess which components of the FRS contribute most to the endpoint Cox regression was repeated with the individual FRS components.

Adjustment was performed for potential confounders, defined as known risk factors of chronic graft failure in RTR, namely: Hba1c and concentration of insulin, primary kidney disease, hs-CRP as biomarker of inflammatory load, use of proliferation inhibitors, use of calcineurin inhibitors, the daily dose of prednisolone, time on haemodialysis pre-transplantation, post-transplant oliguria, proteinuria, time between renal transplantation and inclusion, type of renal transplant (living/dead), Class I HLA antibodies, Class II HLA antibodies, acute rejection and estimated glomerular filtration rate (eGFR). Factors that are included in the calculation of the FRS were not adjusted for.

The contribution of the FRS to risk prediction was assessed. ROC curves were computed for the TVC of the FRS, eGFR and a combined model of both the eGFR and FRS in order to assess the prognostic value of these variables for chronic graft failure. Considering the time-varying aspect of the Framingham risk score, the ROC curves were made with the FRS as a function of time. Furthermore, due to the nested nature of the analysis, the addition of the FRS to the eGFR was assessed using likelihood ratio statistics.

A *p*-value of <0.05 is considered statistically significant. In order to avoid a type-1 error *p*-values were corrected using the Bonferroni-Holm method [20]. All statistical analyses were performed using Stata version 15.

## 3. Results

In this prospective longitudinal study, the FRS was assessed as a potential clinical tool for predicting graft failure in 600 renal transplant recipients. A total of 81 patients (14%) experienced chronic graft failure during the 10-year follow-up. Graft failure occurred due to chronic allograft dysfunction in 50% of patients, chronic allograft nephropathy in 39%, return of primary disease in 4%, acute rejection in 2% and a vascular event in 2%. In 3% of patients, the cause of graft failure was unknown. During the follow-up period, 128 patients (21.3%) died.

Analyses were conducted by dividing patients into the predefined FRS categories of low (<10%, *n* = 155), medium (10–20%, *n* = 158), and high (≥20%, *n* = 287) risk, and differences between baseline characteristics were assessed (Table 1). As expected, all parameters included in the FRS behaved consistently, with higher risk in increasing risk groups (Supplemental Table S1). Additionally, the levels of glucose, HbA1c, and use of statins increased significantly with a higher FRS (*p* < 0.001), likely also due to the inclusion of diabetes and total cholesterol as parameters in the FRS. A high body weight, BMI and higher levels of hs-CRP were also associated with a higher FRS (*p* < 0.001). The number of patients using proliferation inhibitors was highest in the low-risk group (*p* = 0.021). Graft function declined with increasing FRS, though this association was stronger for eGFR (*p* < 0.001) than for creatinine clearance (*p* = 0.02).

**Figure 1:**
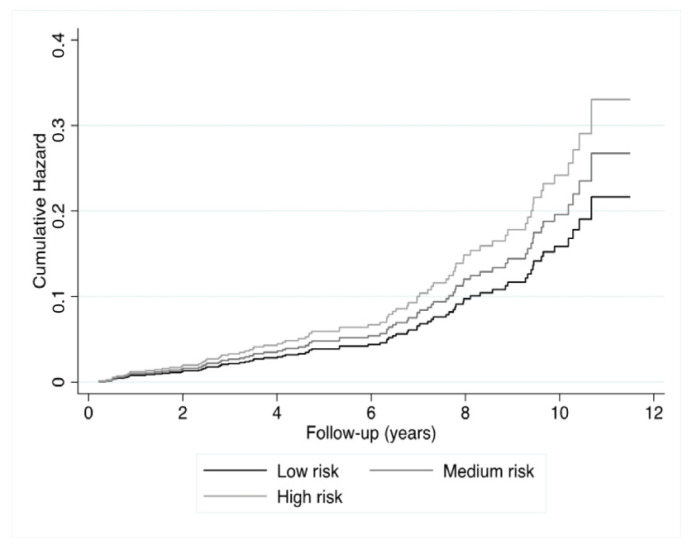
Cumulative hazard ratios of chronic graft failure according to low, high and medium risk of the FRS.

Since the Schoenfeld Residuals test was significant (*p* = 0.003), a multivariable extended Cox model was carried out, including a TVC of the FRS. Cumulative hazard ratios showed that participants with a higher FRS experienced a greater incidence of graft loss (Figure 1, *p* = 0.001). A univariable extended Cox regression (Table 2) demonstrated a strong and highly significant association between FRS (in percent) and graft failure (hazard ratio (HR): 1.04, 95%CI: 1.02–1.06, *p* < 0.001, model 1), indicating a 4% increased risk of developing graft failure over the course of 10 years, per percentage increase in the FRS. The HR of the TVC was 0.99 (95%CI; 0.99–1.00, *p* = 0.002), indicating that the risk decreases by 1% per year. Adjusting for Hba1c and concentration of insulin (model 2, HR: 1.03, 95%CI 1.01–1.05 *p* = 0.001), the underlying primary kidney disease (model 3), hs-CRP (model 4) or use of calcineurin inhibitors, proliferation inhibitors and daily prednisolone dose (model 5) did not considerably alter this association. Adjusting for time on haemodialysis, proteinuria, time between transplantation and baseline, acute rejection, type of renal transplantation Class I HLA antibodies, Class II HLA antibodies and post-transplantation duration of oliguria (model 6, HR: 1.03, 95%CI 1.01–1.05 *p* = 0.001) or eGFR (model 7, HR: 1.03, 95%CI 1.01–1.05), *p* < 0.001) did also not considerably alter the association. The association of the FRS with chronic graft failure remained significant in a fully adjusted model (HR: 1.02, 95%CI 1.00–1.05), *p* = 0.040). These results show that even when considering a substantial number of prevailing risk factors, the FRS remained to be prospectively associated with graft failure. Furthermore, additional analysis showed that patient age, systolic blood pressure, smoking and concentration of HDL-C contributed most to the association of the FRS with chronic graft failure (Supplemental Table S2).

Hazard ratios were obtained using Cox proportional hazard regressions. Groups were according to pre-defined cut-off values of the FRS, namely “low risk” (<10%), “intermediate risk” (10–20%) and “high risk” (>20%).

**Figure 2:**
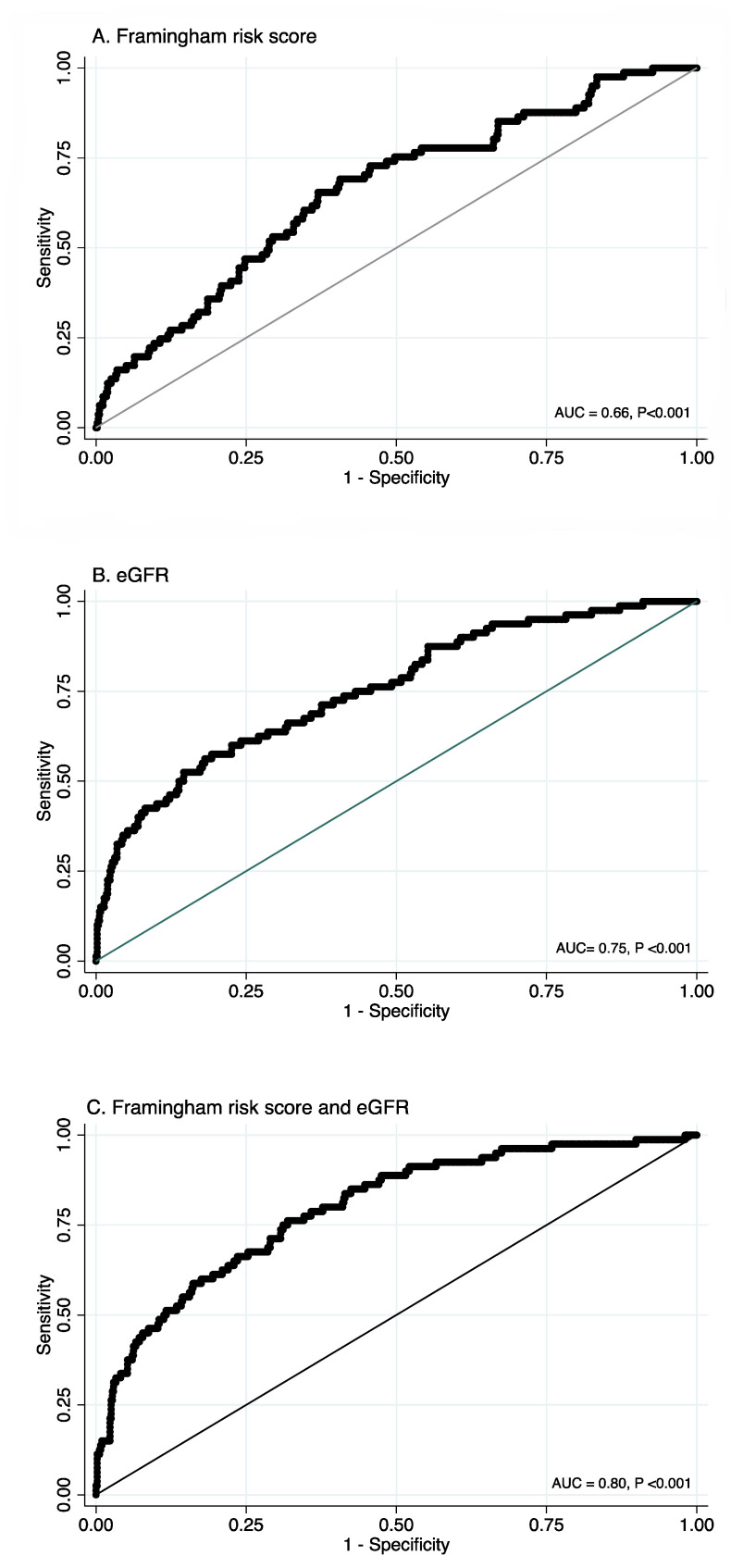
Receiver operating curves.

Next, ROC curves for the FRS, eGFR, and a combination of eGFR and FRS were computed. The FRS ROC curve showed a fair degree of separability, and can therefore serve as a suitable predictor for graft failure (AUC = 0.66, *p* < 0.001, Figure 2A). eGFR is currently the standard biomarker in clinical use and indeed its ROC curve also showed a good degree of separability, and a larger area under the ROC curve (AUC = 0.75, *p* < 0.001, Figure 2B). Combining the FRS and eGFR ROC curves suggested that the FRS would add to the predictive value of eGFR (AUC = 0.79, *p* < 0.001, Figure 2C). Furthermore, when adding the FRS to eGFR the model likelihood ratio statistic increases from 67.60 to 88.39, with a highly significant likelihood-ratio test (*p* < 0.001). These results indicate that using the FRS together with eGFR significantly improves risk prediction of chronic graft failure.

Receiver operating curves for A. the Framingham risk score, B. eGFR and C. the Framingham risk score and eGFR combined. Abbreviations: eGFR, estimated glomerular filtration rate; AUC, area under the curve.

## 4. Discussion

Our results show that the 10-year cardiovascular FRS is significantly associated with chronic graft failure. Although chronic graft failure is driven by a combination of different insults, de novo atherosclerosis manifesting as TV is believed to be a significant contributor. The data indicate that every percent increase in the FRS leads to a 4% increased risk of developing chronic graft failure over the course of 10 years. In the adjusted model, taking all of the most common risk factors into account this association drops to 3%. Furthermore, the hazard decreases by 1% every year. When combining eGFR and the FRS in a ROC curve, the predictive value increases substantially.

Currently, there is no consensus on clinical risk prediction for chronic graft failure in RTR. There have been studies that looked at a variety of potentially useful predictors of graft failure such as serum creatinine, eGFR, proteinuria, acute rejection, acute tubular necrosis, carotid-femoral pulse wave velocity, and use of immunosuppressants. However, most of the conducted research is not readily clinically implementable [21].

A major contributing factor to chronic graft failure is transplant vasculopathy, where lesions closely resembling classical atherosclerosis damage the vascular make-up of the transplanted graft [6,7]. The FRS is, to date, the best-researched CVD risk prediction model and is used worldwide. It includes the risk factors gender, age, smoking, systolic blood pressure, total cholesterol, and HDL-C, all key metrics thought to pathophysiologically contribute to the formation of atherosclerotic plaques [11,22]. Hypothetically, the FRS could be an adequate fit for chronic graft failure risk stratification, an assumption confirmed by the present study.

A well-established risk factor to predict chronic graft failure is eGFR [23]. For this reason, it is interesting that adding the FRS to eGFR as a predictor for chronic graft failure resulted in an even stronger predictive model. It would be recommended to replicate this study in larger, ideally multi-centric cohorts and to integrate eGFR in the FRS. With respect to other potential limitations of this study, it is important to consider the homogeneity of the population. This study was conducted in a single centre in the Northern part of the Netherlands, and all participants were Caucasian. However, the Framingham heart study was also primarily carried out in an all-Caucasian North American city, therefore making it applicable to the study population. Another limitation is that the description of the causes of graft failure available in our study has not been carried out in great detail and did not adhere to the contemporary Banff classification [24] due to the time of inclusion of the participants. The time period of inclusion also entailed this study to have relatively young kidney donors, which does not entirely reflect the current situation with increasing transplantation from older donors. However, kidneys of older patients with more co-morbidities are more likely to already have experienced priming by exposure to atherosclerotic risk factors, potentially making risk stratification by the FRS even more relevant. Therefore, we still believe that our results are relevant to current clinical practice. Furthermore, although our work included enough renal transplant recipients to be sufficiently powered, the number of patients which experienced graft failure was somewhat limited. However, still the data reveal a strong enough association to implicate clinical value.

An advantage of using the FRS is that, in general, all components included in the FRS are susceptible to pharmacotherapy. The dependency of sustained graft functioning on the FRS indicates that, to a certain extent, modifiable risk factors underlie chronic graft failure. Intervention trials would be required to confirm such reasoning. Proper risk stratification has the potential to extend the average 10-year survival of renal grafts, putting less patients in need to consequently being re-transplanted, thereby overall contributing to an increased availability of donor kidneys.

Taken together, the results of this study demonstrate that the FRS has clinical potential as a predictive tool to identify renal transplant recipients at risk of chronic graft failure. Due to the low costs and broad availability of the measurements included in the FRS, implementing the use of the FRS in daily clinical practice is realistic. Effective risk stratification combined with the potential for therapeutic intervention could conceivably contribute to prolonged survival of renal donor grafts.

## Figures and Tables

**Table 1:** Baseline characteristics according to low, medium and high Framingham risk score.

Characteristics	Framingham Risk Score Groups Low (<10%), Medium (10–20%), High (≥20%)			
	Low (*n* = 148)	Medium (*n* = 151)	High (*n* = 301)	*p* Value
**Framingham risk score**				
FRS	5.2 (3.3–7.5)	14.5 (12.3–16.9)	37.5 (26.8–49.5)	<0.001
Age, years	37.4 (31.5–44.1)	49.8 (42.8–55.5)	59.7 (53.0–64.8)	<0.001
Male gender, *n* (%)	58 (39.2)	80 (53.0)	194 (64.4)	<0.001
Smoking, *n* (%)	58 (39.2)	80 (53.0)	194 (64.5)	<0.001
Diabetes, *n* (%)	5 (3.4)	13 (8.6)	84 (27.9)	<0.001
Total cholesterol, mg/dL	204.2 (181.9–225.8)	209.2 (187.6–232.1)	225.1(199.5–249.4)	<0.001
HDL cholesterol, mg/dL	44.3 (35.6–52.8)	40.2 (34.4–50.3)	39.1 (31.7–48.3)	<0.001
Mean systolic blood pressure, mmHg	134 (125–146)	145 (133–156)	162(149–176)	<0.001
Use of antihypertensive medication, *n* (%)	110 (74.3%)	129 (85.4%)	284 (94.4%)	<0.001
**Recipient characteristics**				
Weight, kg	70.0 (61.5–79.5)	76.5 (67.5–87.0)	79.0 (71.0–88.0)	<0.001
BMI, kg/m^2^	23.7 (21.6–27.0)	25.4 (23.0–29.1)	26.4 (24.0–28.7)	<0.001
Use of statins, *n* (%)	52 (35.1)	82 (54.3)	160 (53.2)	<0.001
**Cardiovascular disease history**				
History of MI, *n* (%)	9 (6.2)	12 (8.0)	27 (9.0)	0.60
History of TIA/CVA, *n* (%)	7 (4.8)	7 (4.6)	19 (6.3)	0.69
**Glucose homeostasis**				
Glucose, mmol/L	4.3 (4.0–4.8)	4.5 (4.1–4.8)	4.7 (4.2–5.5)	<0.001
Insulin, μmol/L	10.6 (8.2–14.3)	11.9 (8.1–16.9)	11.2 (7.8–16.1)	0.33
HbA1c, %	5.8 (5.4–6.4)	6.3 (5.9–6.7)	6.7 (6.1–7.5)	<0.001
Use of anti-diabetic drugs, *n* (%)	3 (2.0)	9 (6.0)	63 (20.9)	<0.001
**Inflammation**				
hsCRP, mg/L	1.3 (0.6–3.8)	2.1 (0.7–4.6)	2.2 (1.1–5.9)	<0.001
**Donor demographics**				
Age, years	35.0 (21.0–47.5)	37.0 (23.0–49.0)	40.0 (24.0–51.0)	0.14
Male sex, *n* (%)	81 (55.1)	85 (57.0)	158 (52.5)	0.64
Living kidney donor, *n* (%)	37 (25.0)	21 (13.9)	25 (8.3)	<0.001
**(Pre)transplant history**				
No haemodialysis	15 (10.1)	13 (8.6)	20 (6.6)	0.42
Dialysis time, months	26.5 (12.5–47.0)	26.0 (14.0–51.0)	28.0 (14.0–50.0)	0.65
Number of transplantations	1.0 (1.0–1.0)	1.0 (1.0–1.0)	1.0 (1.0–1.0)	0.61
HLA mismatch	1.00 (0.00, 2.00)	1.00 (0.00, 2.00)	1.00 (0.00, 2.00)	0.58
Class I HLA antibodies	3 (1–6)	3 (1–7)	3 (1–6)	0.20
Class II HLA antibodies	2 (0–5)	2 (1–5)	2 (0–5)	0.07
Acute rejection, *n* (%)	72 (48.65%)	70 (46.36%)	127 (42.19%)	0.39
Time between Tx and inclusion, years	5.6 (2.0–9.2)	6.1 (3.1–12.0)	6.4 (2.7–11.7)	0.11
**Primary renal disease, *n* (%)**				
Primary glomerular disease	42 (28.4)	50 (33.1)	77 (25.6)	0.24
Glomerulonephritis	18 (12.2)	7 (4.6)	14 (4.7)	0.006
Tubulo-interstitial disease	33 (22.3)	26 (17.2)	33 (11.0)	0.006
Polycystic renal disease	12 (8.1)	30 (19.8)	63 (20.9)	0.002
Dysplasia and hypoplasia	11 (7.4)	4 (2.7)	6 (2.0)	0.01
Renovascular disease	5 (3.4)	8 (5.3)	20 (6.6)	0.36
Diabetic nephropathy	3 (2.0)	4 (2.7)	16 (5.3)	0.16
Other or unknown cause	24 (16.2)	22 (14.6)	72 (23.9)	0.03
**Immunosuppressive medication**				
Daily prednisolone dose, mg/dL	10.0 (7.5–10.0)	10.0 (7.5–10.0)	10.0 (7.5–10.0)	0.84
Calcineurin inhibitors, *n* (%)	115 (77.7)	112 (74.2)	244 (81.1)	0.23
Proliferation inhibitors, *n* (%)	118 (80.8)	116 (76.8)	207 (69.2)	0.021
**Renal allograft function**				
Post-transplantation oliguria, days	1.1 (3.4)	1.9 (4.7)	2.9 (8.9)	<0.001
Creatinine clearance mL/min/1.73 m^2^	65.0 (22.3)	64.3 (23.7)	59.6 (21.8)	0.02
eGFR, mL/min	51.8 (40.2–61.9)	47.7 (36.8–58.6)	44.9 (34.0–55.8)	<0.001
Proteinuria ≥ 0.5 g/24 h, *n* (%)	39 (26.4)	39 (26.0)	90 (30.0)	0.58
Graft failure, *n* (%)	24 (16.2)	23 (15.2)	34 (11.3)	0.28

Normally distributed continuous variables are presented as mean ± SD, and differences were tested with one-way analysis of variance (ANOVA). Continuous variables with a skewed distribution are presented as median (25th to 75th percentile), and differences were tested by Kruskal–Wallis test. Categorical data are summarised as *n* (%), and differences were tested by chi-squared test. Abbreviations: FRS, Framingham risk score; HDL, high density lipoprotein; BMI, body mass index; MI, myocardial infarct; TIA, transient ischaemic attack; CVA, cerebrovascular accident; HbA1c, haemoglobin A1c; hs-CRP, high sensitivity C-reactive protein; HLA, human leukocyte antigens; eGFR, estimated glomerular filtration rate.

**Table 2:** Hazard ratios for chronic graft failure per one percent increase of the Framingham risk score.

	Hazard Ratio Main Effect	95%CI	*p* Value	*p* Value after Bonferroni-Holm Correction	Hazard Ratio TVC	95%CI	*p* Value	*p* Value after Bonferroni-Holm Correction
Model 1	1.04	1.02–1.06	<0.001	0.008	0.99	0.99–1.00	0.002	0.016
Model 2	1.03	1.01–1.05	0.001	0.008	0.99	0.99–1.00	0.002	0.016
Model 3	1.04	1.02–1.06	<0.001	0.008	0.99	0.99–1.00	0.002	0.016
Model 4	1.04	1.02–1.06	<0.001	0.008	0.99	0.99–1.00	0.002	0.016
Model 5	1.04	1.02–1.06	<0.001	0.008	0.99	0.99–1.00	0.002	0.016
Model 6	1.03	1.01–1.05	<0.001	0.001	0.99	0.99–1.00	0.002	0.016
Model 7	1.03	1.01–1.05	0.006	0.012	0.99	0.99–1.00	0.002	0.016
Model 8	1.02	1.00–1.05	0.040	0.012	0.99	0.99–1.00	0.008	0.016

Model 1: crude; model 2: adjustment for Hba1c and concentration of insulin; model 3: adjustment for primary kidney disease; model 4: adjustment for C-reactive protein concentration; model 5: adjustment for use of calcineurin inhibitors, proliferation inhibitors, and dose of prednisolone; model 6: adjustment for time on haemodialysis, proteinuria, time between renal transplant and baseline, acute rejection, type of renal transplantation, Class I HLA antibodies, Class II HLA antibodies and post-transplantation duration of oliguria; model 7: adjustment for eGFR; model 8: fully adjusted. Abbreviations: CI, confidence interval; TVC, time varying covariate.

## Data Availability

The data that support the findings of this study are available from the corresponding author upon reasonable request.

## Supplementary Materials

The following are available online at https://www.mdpi.com/article/10.3390/jcm10153287/s1, Table S1: Number of patients at risk per risk group of the FRS; Table S2: Association of individual components of the FRS with chronic graft failure.
