# Supplementary material for: The Framingham Risk Score Is Associated with Chronic Graft Failure in Renal Transplant Recipients
Source: J Clin Med. 2021 Jul 26;10(15):3287. doi: 10.3390/jcm10153287 (PMC8348129; doi:10.3390/jcm10153287)
Supplement: Supplementary file 1 [file jcm-10-03287-s001.zip › jcm-1283868-supplementary.pdf]

Supplemental Content

Supplemental Table S1. Number of patients at risk per risk group of the FRS.

Years of Follow Up	Low Risk	Medium Risk	High Risk
0	147	151	300
2	141	145	271
4	137	135	239
6	130	127	199
8	119	113	167
10	93	84	113
12	0	0	0

Supplemental Table S2. Association of individual components of the FRS with chronic graft failure.

	Hazard Ratio	95% CI	*p*-Value
Age	0.97	0.95-0.99	0.006
Sex	0.98	0.62-1.55	0.92
Systolic blood pressure	1.02	1.00-1.02	0.002
Smoking	1.92	1.21-3.06	0.006
Use of anti-hypertensives	1.02	0.50-2.08	0.95
Diabetes	1.19	0.64-2.20	0.58
Concentration of HDL-C	0.97	0.94-0.99	0.003
Concentration of total cholesterol	1.00	1.00-1.01	0.41

Hazard ratios are computed by Cox proportional hazard regression. Abbreviations: HDL-C, high density lipoprotein cholesterol.
